# Finite Element Analysis of Aluminum Honeycombs Subjected to Dynamic Indentation and Compression Loads

**DOI:** 10.3390/ma9030162

**Published:** 2016-03-04

**Authors:** A.S.M. Ayman Ashab, Dong Ruan, Guoxing Lu, Arafat A. Bhuiyan

**Affiliations:** Faculty of Science, Engineering and Technology, Swinburne University of Technology, Hawthorn, VIC 3122, Australia; aashab@swin.edu.au (A.S.M.A.A.); glu@swin.edu.au (G.L.); abhuiyan@swin.edu.au (A.A.B.)

**Keywords:** finite element analysis, indentation, relative density, tearing energy, strain rate

## Abstract

The mechanical behavior of aluminum hexagonal honeycombs subjected to out-of-plane dynamic indentation and compression loads has been investigated numerically using ANSYS/LS-DYNA in this paper. The finite element (FE) models have been verified by previous experimental results in terms of deformation pattern, stress-strain curve, and energy dissipation. The verified FE models have then been used in comprehensive numerical analysis of different aluminum honeycombs. Plateau stress, *σ_pl_*, and dissipated energy (*E_I_* for indentation and *E_C_* for compression) have been calculated at different strain rates ranging from 10^2^ to 10^4^ s^−1^. The effects of strain rate and *t/l* ratio on the plateau stress, dissipated energy, and tearing energy have been discussed. An empirical formula is proposed to describe the relationship between the tearing energy per unit fracture area, relative density, and strain rate for honeycombs. Moreover, it has been found that a generic formula can be used to describe the relationship between tearing energy per unit fracture area and relative density for both aluminum honeycombs and foams.

## 1. Introduction

Over the last few decades, man-made honeycombs have been widely used in many industries due to their properties such as high strength to weight ratio and good energy absorption capabilities. Honeycombs are manufactured from materials such as aluminum, nomex, polymer, and ceramic. Aluminum honeycombs can be used as industrial products as well as core materials in sandwich panels in various fields of engineering such as aerospace, aircraft, automotive, and naval engineering [1,2]. 

A number of studies have been conducted on the out-of-plane compression of aluminum honeycombs at low and intermediate strain rates [3,4,5,6,7,8]. Zhou and Mayer [3], Wu and Jiang [4] and Baker *et al.* [5] conducted compression tests on aluminum honeycombs at different strain rates in the out-of-plane direction and found that the plateau stress, *σ_pl_*, increased with strain rate, $\dot{\varepsilon }$. Both Xu *et al.* [6] and Ashab *et al.* [7] found that with the increase of *t/l* ratio (cell wall thickness to edge length ratio) and strain rate, plateau stress, *σ_pl_*, increased. Vijayasimha Reddy *et al.* [8] concluded that energy absorption capacity of aluminum honeycombs increased with the impact velocity under out-of-plane compression load. Alavi and Sadeghi [9] conducted experiments on foam-filled aluminum hexagonal honeycombs under the out-of-plane compression loads. They observed that the crushing strength of bare honeycombs and foam-filled honeycombs increased with strain rate and bare honeycombs were more sensitive to strain rate than foam-filled honeycombs. Mozafari *et al.* [10] employed ABAQUS software and observed that the mean crushing strength and energy absorption of foam-filled honeycomb were greater than the sum of those of bare honeycomb and foam. 

Along with the experimental investigation, finite element analysis (FEA) has also been conducted by various researchers [11,12,13,14] to study the mechanical behavior of aluminum honeycombs. Guo and Gibson [11] conducted numerical analysis of intact and damaged honeycomb properties in the in-plane direction and reported that modulus and strength decreased due to the effect of single and isolated defects of various sizes. They also investigated the separation distance between two defects and its effect on the plastic collapse strength and Young’s modulus. Ruan *et al.* [12] employed ABAQUS to investigate the effects of *t/l* ratio and impact velocity on the in-plane deformation mode and plateau stress. They derived an empirical formula to describe the relationship between the plateau stress, *t/l* ratio and velocity. Hu *et al.* [13,14] conducted experiments as well as finite element analysis to study in-plane crushing of aluminum honeycombs. They proposed a dynamic sensitivity index to describe crushing strength and energy absorption. 

Deqiang *et al.* [15] used ANSYS/LS-DYNA [16] to study the out-of-plane dynamic properties of aluminum hexagonal honeycomb cores in compression. They found that the out-of-plane dynamic plateau stresses of honeycombs were related to the impact velocity, *t/l* ratio, and expanding angle *θ* of honeycombs by power laws. Yamashita and Gotoh [17] conducted both experimental and numerical analyses on the compression of aluminum honeycombs. The crushing strength was related to the *t/l* ratio of honeycombs by a power law with the exponent of 5/3, which was the same as the theoretical equation derived by Wierzbicki [18]. The computer simulation carried out by Xu *et al.* [19] also found a power law relationship between the out-of-plane compressive strength of aluminum honeycombs and the strain rate and *t/l* ratio. 

A limited number of experiments were conducted on aluminum honeycombs subjected to indentation [3,7] at very low and intermediate strain rates. Zhou and Mayer [3] conducted quasi-static indentation tests on aluminum honeycombs to study the influence of specimen size on the force *versus* displacement curve. They found flatter and lower indentation force for the larger specimen. This was because larger specimens had a larger amount of surrounding cells, which provided stiffer support and resulted in fewer cells to be involved in tearing. The four primary deformation mechanisms were shear, tearing initiation, tearing, and compression. Zhou and Mayer also used different indenters, such as square, rectangular, and circular, to study the effect of indenter shape. Ashab *et al.* [7] conducted indentation tests on three types of aluminum honeycombs at strain rates from 10 to 10^2^ s^−1^ and found that the tearing energy increased with the *t/l* ratio of honeycomb and strain rate. However, due to the limited honeycombs and testing machines available, previous studies were not able to draw quantitative conclusions on the effects of *t/l* ratio and strain rate on the tearing energy of honeycombs.

In the present paper, numerical simulation is performed using ANSYS/LS-DYNA [16] to study the dynamic out-of-plane properties of aluminum hexagonal honeycombs with various *t/l* ratios subjected to indentation. Compression of honeycombs is also simulated in order to calculate the tearing energy in indentation. Full-scale FE models of honeycombs are verified by the previous experimental results. The verified FE models are then used to investigate the effects of *t/l* ratio and strain rate on the plateau stress and tearing energy of honeycombs subjected to indentation. Empirical equations are proposed. 

## 2. Finite Element (FE) Modeling 

**Figure 1:**
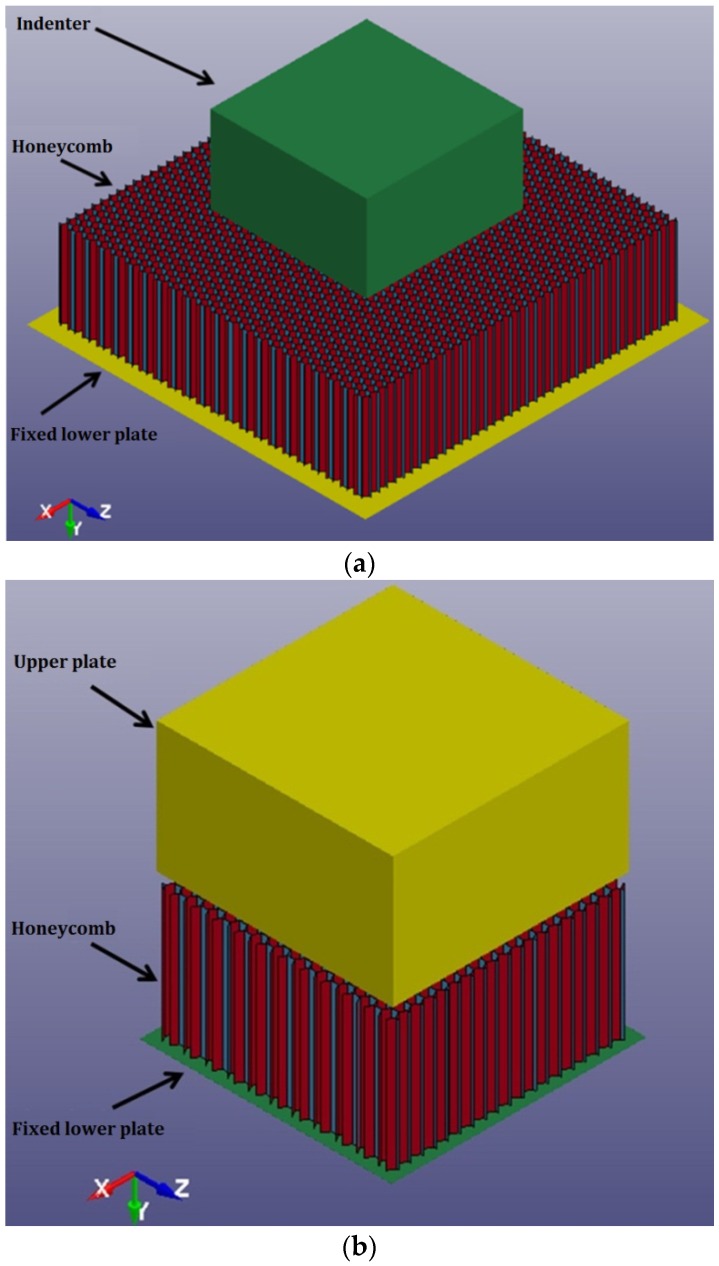
Typical FE models of honeycomb H31: (**a**) indentation; (**b**) compression.

In the present paper, numerical analysis of aluminum honeycombs was carried out using ANSYS/LS-DYNA [16]. Two types of honeycombs, differing in cell size and cell wall thickness, were simulated. The honeycombs are named as H31 and H42 for honeycombs 3.1-3/16-5052-.001N, 4.2-3/8-5052-.003N, respectively. The specifications of the honeycombs, provided by the manufacturer, are listed in Table 1. The dimensions of each honeycomb model are the same as those of the actual specimen used in the previous experiments [7]. The height of all honeycombs, *h*, was 50 mm. The in-plane dimensions of all honeycomb specimens were 180 mm × 180 mm in indentation simulation (Figure 1a) and 90 mm × 90 mm in compression simulation (Figure 1b). 

Aluminum honeycomb walls were simulated using a bilinear kinematic hardening material model. The corresponding material properties are listed in Table 2. Belytschko-Tsay Shell 163 elements with five integration points were employed to simulate the honeycomb cell walls for high computational efficiency [19]. In each honeycomb cell, single wall thickness was employed for the four oblique walls and double wall thickness was employed for the two vertical walls. To identify the optimum element size, a convergence test was carried out. Five different element sizes—2.1 mm, 1.4 mm, 0.7 mm, 0.3 mm, and 0.15 mm—were used to simulate compression of honeycombs at 5 ms^−1^. No significant difference (less than 7%) was observed between the results for element sizes 0.7 mm and 0.15 mm. Therefore, in this FE analysis of aluminum honeycombs an element size of 0.7 mm was employed. Since tearing of cell walls happened in honeycombs under indentation, MAT_ADD_EROSION failure criterion with a maximum effective strain of 0.3 [21] was used in the indentation models. All degrees of freedom of one node at a corner of the honeycomb were fixed to keep the honeycomb in place (*i.e.*, no rigid body movement). 

In physical experiments, honeycomb specimens were placed on a fixed lower plate and crushed by an upper plate (in compression) or indenter (in indentation). In FE models, the lower plate was simulated by a rigid plate while the upper plate and indenter were simulated by rigid bodies. The lower plate was 1 mm (thickness) × 200 mm × 200 mm. The upper plate and indenter were cuboids with dimensions of 50 mm (height) × 90 mm × 90 mm, the same as in the previous experimental study [7]. The material properties used for the plates and indenter are listed in Table 3. 

For the lower plate, all degrees of freedom were fixed. For the upper plate (in compression) and indenter (in indentation), all three rotational movements and two transitional movements in the X and Z directions were fixed. The upper plate or indenter could move in the negative Y direction at a constant velocity to compress or indent honeycombs. 

A tiny gap (0.1 mm) between the fixed lower plate and the honeycomb was employed to avoid the initial penetration at the beginning of the simulation. For the same reason, an initial gap of 5 mm was also introduced between the upper plate or the indenter and the honeycomb. SURFACE_TO_SURFACE contacts were employed between the plates or indenter and honeycomb. Typical finite element models of indentation and compression of honeycombs in the out-of-plane direction are shown in Figure 1.

## 3. Validation of FE Models

### 3.1. Deformation Patterns 

**Figure 2:**
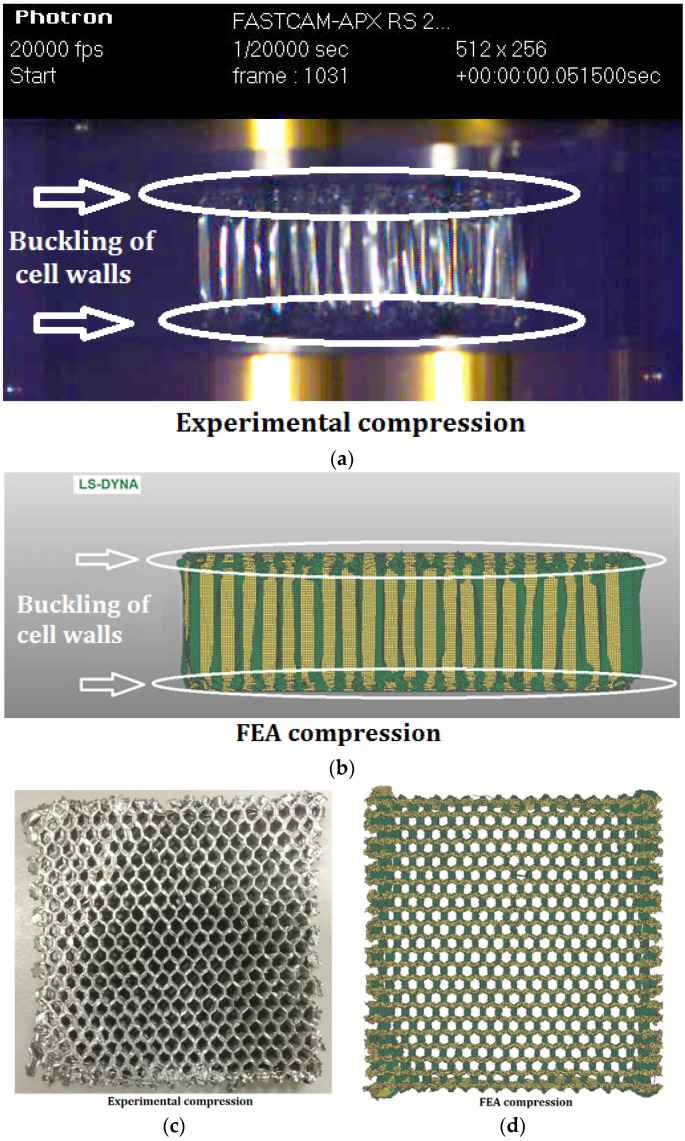
Comparison between experimental and simulated deformation mode of honeycomb H31 under compression: (**a**) experimental result; (**b**) FEA result; (**c**) experimental post-test specimen; (**d**) FEA post-test specimen.

Figure 2 shows comparison between the experimental and simulated deformation of honeycomb H31 in compression at 5 ms^−1^. Identical deformation mode in both the experiments [6,7] and FEA was observed: when the honeycomb was compressed in the out-of-plane (T) direction, buckling of cell walls was initiated from both the top and bottom ends and propagated to the middle of the honeycomb (Figure 2a,b). Figure 2c,d show the deformed honeycomb H31 after crushing in the experiment and FEA, respectively. Almost identical deformation patterns were found in the experimental and FEA results. Due to the stronger lateral constraints in the central part of the honeycomb, the honeycomb deformed in a much more regular pattern in the central part. However, along the four edges of the indenter, honeycomb cell walls deformed in an irregular pattern. Similar deformation patterns were observed for honeycomb H42 in compression.

**Figure 3:**
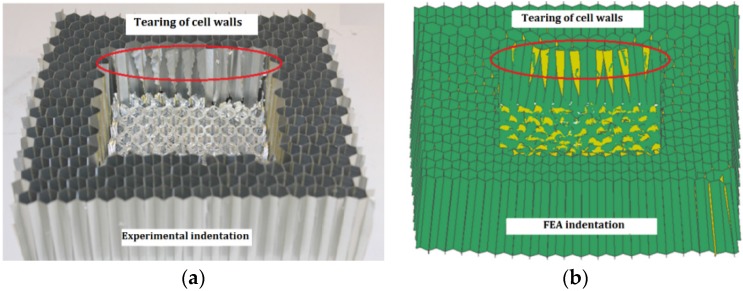
Comparison between experimental [7] and FEA deformation pattern of honeycomb H42 under indentation: (**a**) experimental post-test specimen; (**b**) FEA post-test specimen.

Figure 3 shows a comparison between experimental and FEA deformation patterns of honeycomb H42 subjected to out-of-plane indentation at a velocity of 5 ms^−1^. Similar irregular tearing patterns were observed in both the experiment and FEA. The FEA results of another type of honeycomb, H31, also showed a similar deformation pattern to that observed in the previous experiments [7]. 

### 3.2. Stress-Strain Curves

**Figure 4:**
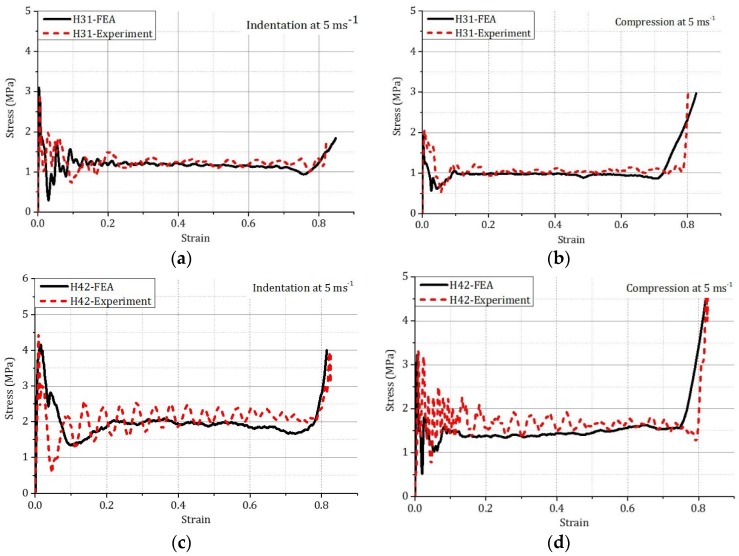
Experimental and FEA stress–strain curves of two types of honeycombs at 5 ms^−1^: (**a**) indentation of H31; (**b**) compression of H31; (**c**) indentation of H42; (**d**) compression of H42.

FEA and experimental stress–strain curves of two types of honeycombs are shown in Figure 4. Similar general trends in the stress-strain curves were found for both honeycombs in indentation and compression.

The plateau stress is defined as the average stress between displacements from 5 to 38 mm. The total dissipated energy is the area under the force-displacement curves up to 38 mm, which is described by *E_C_* in compression and *E_I_* in indentation. Tearing of the cell walls along the four edges of the square indenter occurred simultaneously during the indentation. Tearing energy, *E_t_*, was calculated using the following energy conservation equation:

*E_t_ = E_I_ − E_C_* (1)

where, *E_t_* is the dissipated energy in tearing; *E_I_ is the* energy dissipated in indentation; and *E_C_ is the* energy dissipated in compression.

Comparisons between the FEA and experimental results in terms of plateau stress and dissipated energy are listed in Table 4. For two different types of honeycombs (H31 and H42), the simulated plateau stresses and total dissipated energies were found to be slightly lower than the corresponding experimental values in both indentation and compression. The differences were between 4.71% and 11.62%, which was acceptable. 

## 4. Results and Discussion

### 4.1. The Effect of t/l Ratio

The effect of *t/l* ratio on the mechanical properties of honeycombs is discussed in this section. Firstly, the thickness of honeycomb cell walls was fixed as 0.0254 mm. Five different cell sizes—3.175 mm, 3.969 mm, 4.763 mm, 6.35 mm, and 9.525 mm—were employed. A constant strain rate of 1 × 10^3^ s^−1^ was used in the simulation. The FEA results are listed in Table 5. Both in indentation and compression, it was found that the plateau stress decreased with the increase of cell size for a constant cell wall thickness. Similar to the plateau stress, dissipated energy and tearing energy also decreased with the increase of cell size.

Secondly, honeycomb cell size, *D*, was kept constant at 4.763 mm (the corresponding cell edge length was 2.75 mm). The thickness of the cell wall varied from 0.0178 to 0.1524 mm, where the corresponding *t/l* ratios were from 0.00647 to 0.05542. The simulation results at a constant strain rate of 1 × 10^3^ s^−1^ are listed in Table 6. 

**Figure 5:**
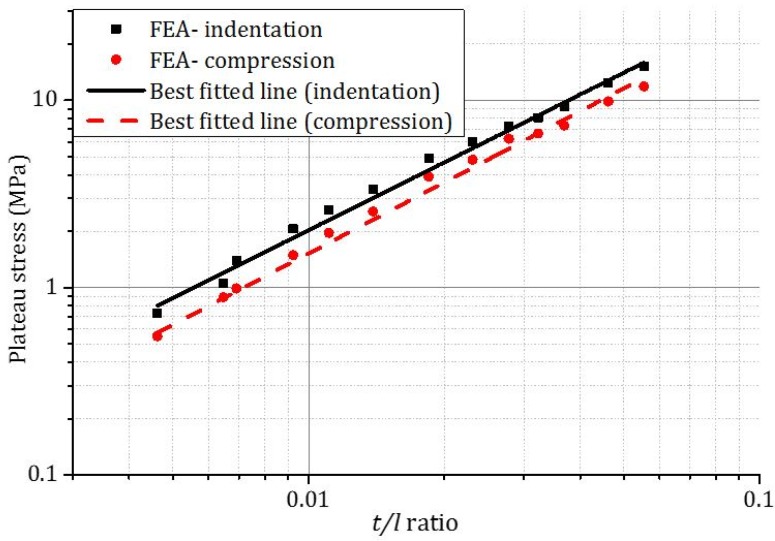
The effect of *t/l* ratio on the plateau stresses of honeycombs under compression and indentation loads at a strain rate of 1 × 10^3^ s^−1^.

The plateau stresses of honeycombs subjected to out-of-plane compression and indentation were found to increase with *t/l* ratio (Figure 5) by power laws. The exponents are 1.47 for compression (Equation (2a)) and 1.36 for indentation (Equation (2b)). Xu *et al.* [6,19] also found a similar power law relation between plateau stress and *t/l* ratio with an exponent of 1.49. 

For compression,

(2a) $$\sigma_{pl}=2.93\sigma_{ys}{\left(t/l\right)}^{1.47}$$

For indentation,

(2b) $$\sigma_{pl}=4.49\sigma_{ys}{\left(t/l\right)}^{1.36}$$

**Figure 6:**
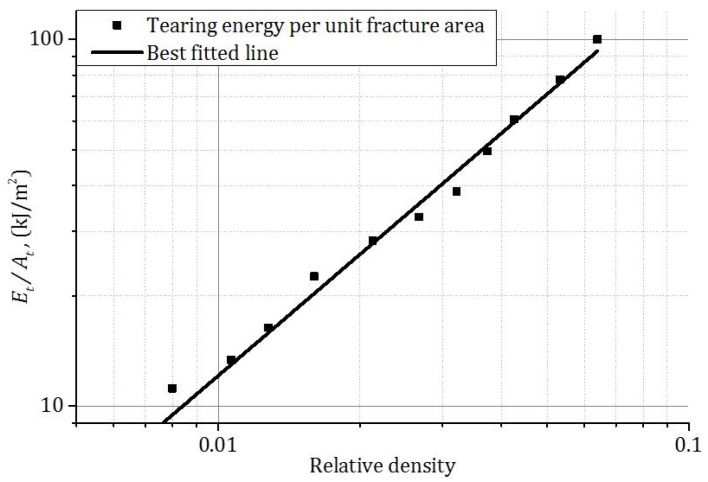
The relationship between the tearing energy per unit fracture area and relative density of honeycomb at a strain rate of 1 × 10^3^ s^−1^.

The tearing energies were calculated using Equation (1) and are shown in Table 5 and Table 6. Tearing energy was also found to increase with the *t/l* ratio. The fracture area, *A_t_*, was calculated as the product of the circumferential length of the square shape indenter (90 mm × 4) and the displacement (38 mm) of the indenter [3]. The relationship between the tearing energy per fracture area, *A_t,_* and the relative density, $\rho /\rho_{0}$, is shown in Figure 6. It was found that with the increase of *t/l* ratio or relative density, the tearing energy per unit fracture area increased. 

**Figure 7:**
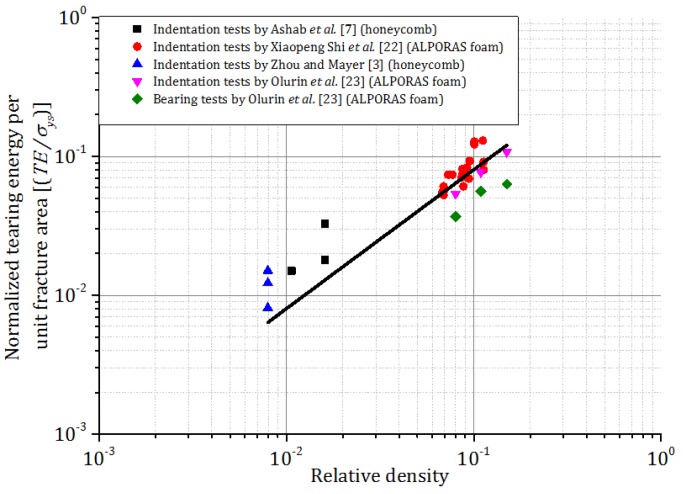
Normalized tearing energy per unit fracture area *vs.* relative density of different cellular materials.

Previously, Zhou and Mayer [3] and Ashab *et al.* [7] conducted quasi-static indentation tests on different honeycombs. Moreover, other researchers [22,23,24] conducted quasi-static indentation tests on aluminum foams. Shi *et al.* [22] proposed a theoretical formula and an empirical formula between tearing energy per unit fracture area and relative density. In order to compare these two types of cellular materials (honeycomb and foam, which are made from different aluminum alloys), tearing energy per unit fracture area was normalized by the yield stress of the parent aluminum alloy for both honeycombs and foams. The relationship between the normalized tearing energy per unit fracture area and relative density is shown in Figure 7. Using yield stress *σ_ys_* = 150 MPa for the foams in Shi *et al.* [22], Olurin *et al.* [23], and Olurin *et al.* [24], the equation proposed by Shi *et al.* [22], $\gamma =119.4\bar{\rho }$, can be rewritten as $\gamma =0.79\sigma_{ys}\bar{\rho }$, where γ, $\sigma_{ys\;}$, and $\bar{\rho }$ are tearing energy per unit area, yield stress of aluminum, and relative density of foam, respectively. The normalized tearing energy per unit fracture area for both aluminum foams and honeycombs are plotted together in terms of relative density in Figure 7. The equation of the best fitted line is as follows, which is very similar to that for foams:

(3) $$\frac{E_{t}}{A_{t}}=0.80\sigma_{ys}(\rho /\rho_{0})$$

### 4.2. The Effect of Strain Rate, $\dot{\varepsilon }$

#### 4.2.1. Plateau Stress

**Figure 8:**
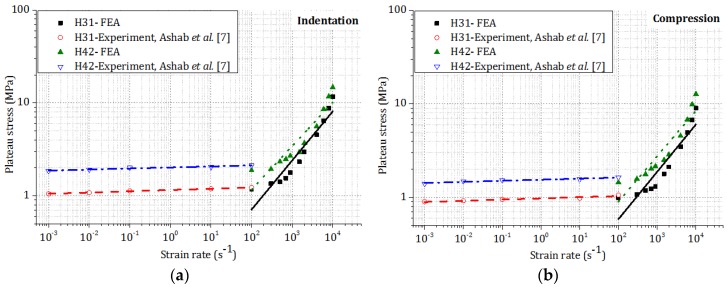
Effect of strain rate on the plateau stresses of two types of honeycombs subjected to: (**a**) indentation; (**b**) compression.

In the previous experimental study [7], honeycombs were crushed at low and intermediate strain rates (1 ×10^−3^ to 1 × 10^2^ s^−1^). FEA was conducted on honeycombs at high strain rates (1 × 10^2^ to 1 × 10^4^ s^−1^). Both experimental and FEA results are shown in Figure 8, which demonstrates the influence of strain rate on the plateau stress of two different honeycombs subjected to out-of-plane indentation and compression loadings, respectively. For both types of honeycombs, the plateau stress increased with strain rate in both indentation and compression. Due to the higher *t/l* ratio, the plateau stress is larger for honeycomb H42 than that for honeycomb H31. 

**Figure 9:**
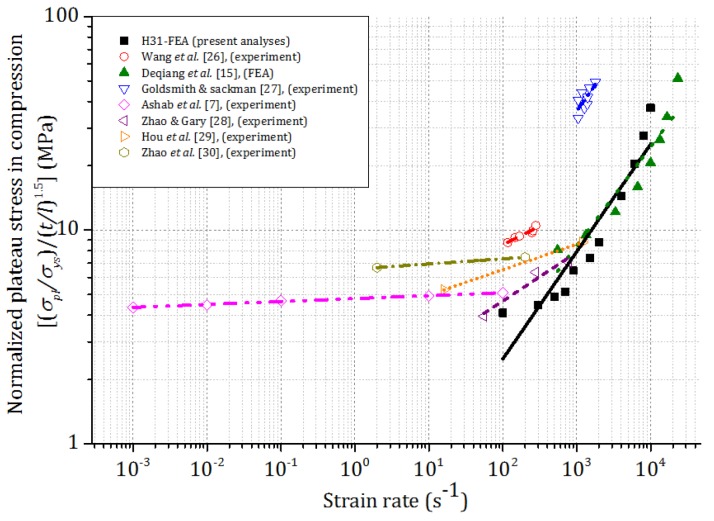
Normalized plateau stress of honeycomb *vs.* strain rate of honeycombs in compression.

Experiments and FEA of compression of aluminum honeycombs were conducted by various researchers [7,15,25,26,27,28,29,30]. In previous experimental study [7], enhancement in the plateau stress was observed at low and intermediate loading velocities. Wang *et al.* [26] reported remarkable enhancement of plateau stress at high impact velocity (20–80 ms^−1^). Goldsmith and Sackman [27] found a 50% enhancement in plateau stress at dynamic velocities up to 35 ms^−1^. Zhao and Gary [28] observed significant enhancement (approximately 40%) in the plateau stress when the loading velocity increased from quasi-static to dynamic (2–28 ms^−1^). Similar enhancement of plateau stress with the loading velocity was also discussed by Hou *et al.* [29] and Zhao *et al.* [30]. In order to compare these results with the current FEA, plateau stresses of honeycombs were normalized as (*σ_pl_/σ_ys_)/(t/l)*^1.5^ and plotted in Figure 9 in terms of strain rate. These current FEA results show significant enhancement of plateau stress at high impact velocities, which agree very well with the FEA results of Deqiang *et al.* [15]. 

#### 4.2.2. Energy Dissipation

**Figure 10:**
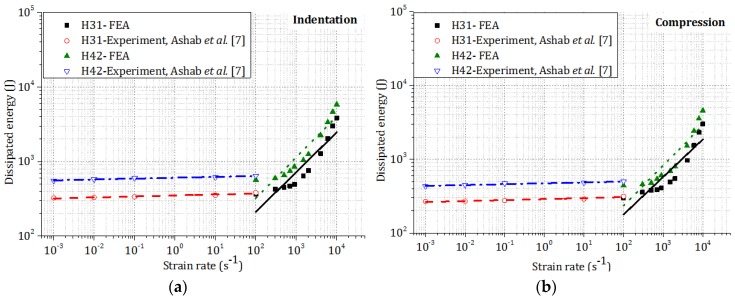
Effect of high strain rate on the total dissipated energy of two types of honeycombs: (**a**) indentation; (**b**) compression.

Figure 10 shows the effect of strain rate on the dissipated energy of two types of honeycombs under indentation and compression loadings, respectively. Similar to the plateau stress, for two types of honeycombs the dissipated energy increased with strain rate in both indentation and compression. For honeycomb H42, the dissipated energies in both indentation and compression were found to be larger than those of honeycomb H31 due to the higher *t/l* ratio. 

**Figure 11:**
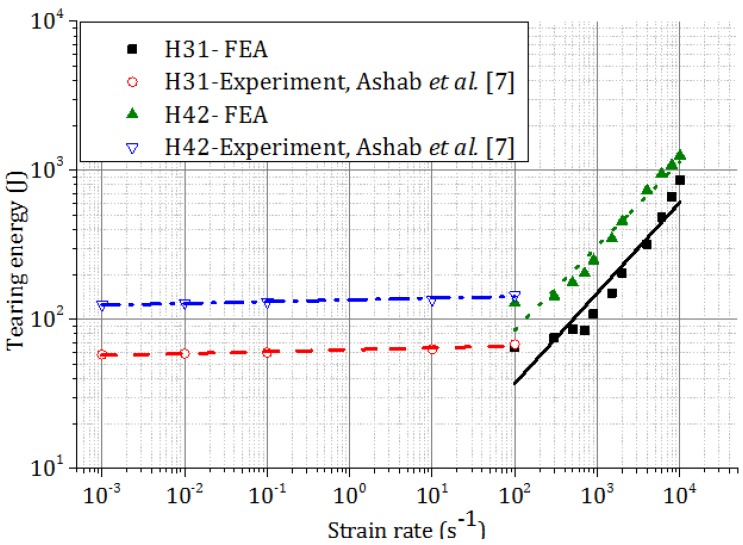
Effect of strain rate on the tearing energy of different honeycombs.

**Figure 12:**
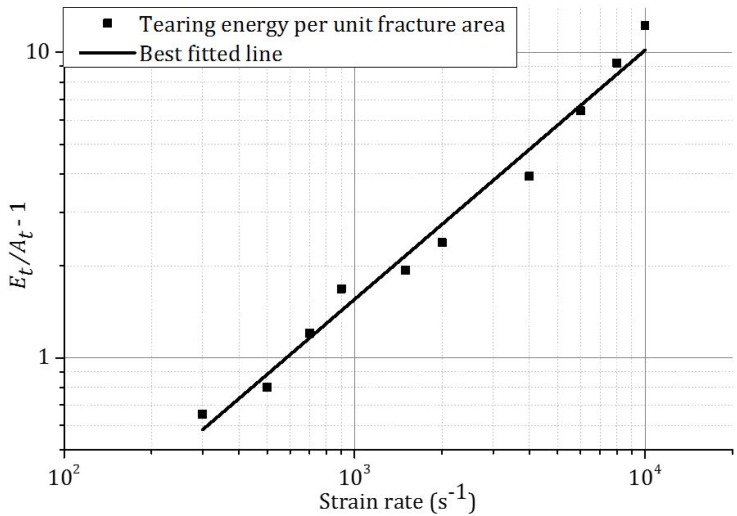
The dependency of tearing energy per unit fracture area of honeycombs and strain rate.

Tearing energy, which is the difference between the total energies dissipated in indentation and compression, was plotted in Figure 11. Due to the higher *t/l* ratio, the magnitude of tearing energy is larger for honeycomb H42 than that for honeycomb H31 at the same strain rate. For both honeycombs, tearing energy increases with strain rate. The fitted curve for tearing energy per unit fracture area for honeycombs at different strain rates is shown in Figure 12. 

The relation between the tearing energy per unit fracture area and the relative density and strain rate is described by the following equation:

(4) $$\frac{E_{t}}{A_{t}}\;=1.37\times 10^{3}{\left(\rho /\rho_{0}\right)}^{1.32}\left(1+8.77\times 10^{-4}{\dot{\varepsilon }}^{1.03}\right)$$

### 4.3. Deformation Pattern of Aluminum Honeycombs Subjected to Compression and Indentation

**Figure 13:**
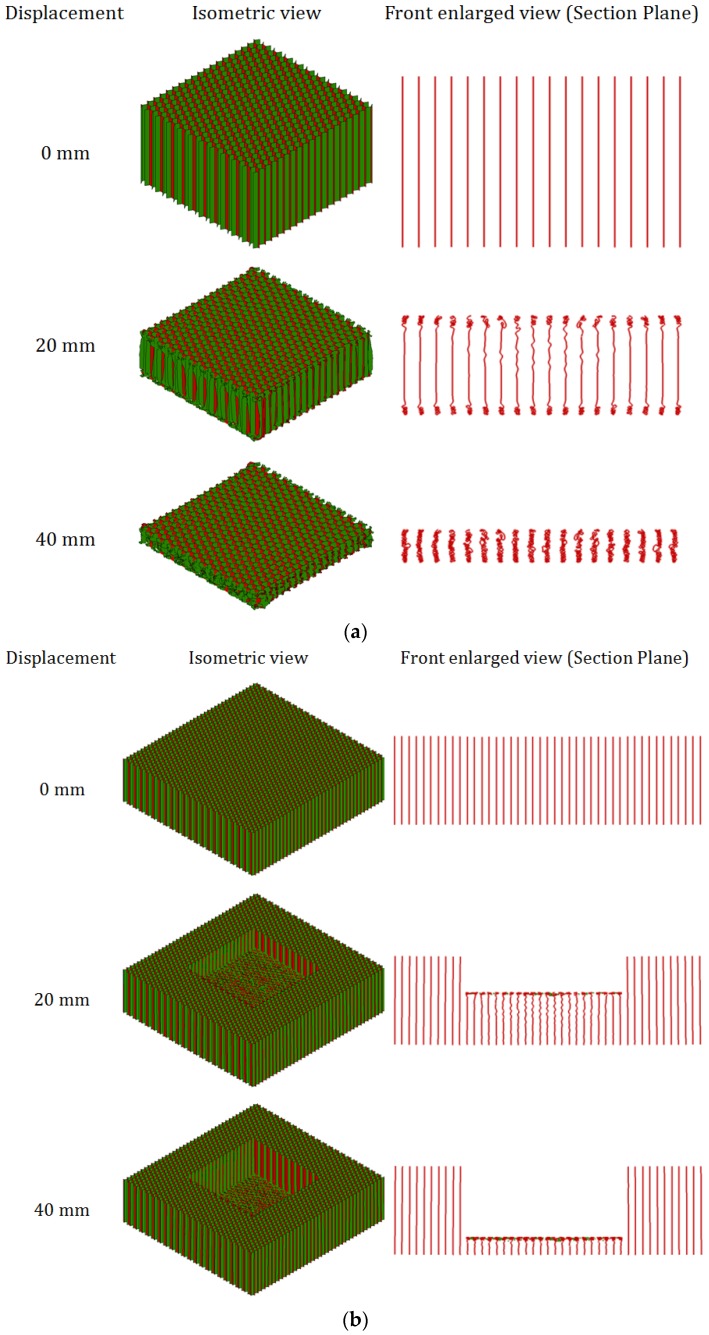
Deformation of honeycomb H31 at 5 ms^−1^: (**a**) compression; (**b**) indentation.

Figure 13 shows the enlarged isometric and front (sectional plane) views of honeycombs H31 under out-of-plane indentation and compression loads. Three images of deformation were taken at a displacement of 0 mm, 20 mm, and 40 mm, respectively, from the animation of FEA by using LS-Prepost software [31]. In Figure 13a it is seen that the progressive buckling of the cell wall occurs from both ends of the honeycomb simultaneously and propagates to the middle region of the honeycomb, which is similar to that observed in the previous experimental study [7]. Deformation mode is found to be independent of strain rate. Xu *et al.* [6] also observed a negligible effect of strain rate on the buckling of honeycomb cells in the out-of-plane compression.

In the previous experimental study, it was impossible to observe the deformation of honeycomb under the indenter. In the current FEA, the deformation of honeycomb in indentation is observed from the front sectional plane view, as shown in Figure 13b. It is found that the progressive buckling of cell walls initiates from the top end of the honeycomb, which is immediately beneath the indenter, and propagates in the same manner till densification. Progressive buckling takes place in the middle portion of the honeycomb model underneath the indenter, which is associated with the tearing of cell walls along the four edges of the indenter. No significant difference is observed in the buckling pattern at different strain rates.

## 5. Conclusions

In this finite element analysis, different honeycomb models have been developed by using ANSYS/LS-DYNA to study the mechanical behavior of honeycombs under out-of-plane indentation and compression loads over a wide range of high strain rates from 1 × 10^2^ to 1 × 10^4^ s^−1^. The FE models have been validated by the previous experimental results (compression and indentation) in terms of deformation, stress-strain curves, plateau stress, and dissipated energy. A reasonable agreement between the FEA and experimental results has been found for both honeycombs H31 and H42. 

It is found that the plateau stress, dissipated energy, and tearing energy increase with the *t/l* ratio. For a constant strain rate of 1 × 10^3^ s^−1^, the plateau stresses increase with *t/l* ratio by power laws with exponents of 1.47 and 1.36 for compression and indentation, respectively. 

Moreover, the plateau stress, dissipated energy, and tearing energy increase gradually for low and intermediate strain rates. Significant enhancement in the plateau stress, dissipated energy, and tearing energy is observed at high strain rates for honeycombs subjected to either compression or indentation loads. An empirical formula is proposed for the tearing energy per unit fracture area in terms of strain rate and relative density of honeycombs. 

The current FEA reveals that at velocities at 5 ms^−1^, under indentation, plastic buckling of the honeycomb cell walls occurs from the end that is adjacent to the indenter, while under compression the buckling of honeycomb cell walls occurs from both ends of the honeycomb. 

It is found that under quasi-static indentation, the empirical formula proposed by Shi *et al.* for foam can be used for honeycombs as well.

## Figures and Tables

**Table 1:** Specification of aluminum honeycombs [20].

Type	Material Description *	Cell Size, D (mm)	Single Cell Wall Thickness, t (mm)	Cell Wall Thickness to Edge Length Ratio, t/l
H31	3.1-3/16-5052-0.001N	4.763	0.0254	0.00924
H42	4.2-3/8-5052-0.003N	9.525	0.0762	0.0139

* In the material description, 3.1 and 4.2 are the nominal densities in pounds per cubic foot, 3/16 and 3/8 are the cell size in inches, 5052 is the aluminum alloy grade, 0.001 or 0.003 is the nominal foil thickness in inches, and N denotes non-perforated cell walls. Data were provided by the manufacturer, HEXCEL^®^ (Stamford, CT, USA). The relation between cell size, *D* end cell edge length, *l* is: *D = √3l*.

**Table 2:** Material properties used in the FE model of aluminum honeycombs [19].

Material Properties	Mass Density (ρ)	Young’s Modulus (E)	Poisson’s Ratio (υ)	Yield Stress (σ_ys_)	Tangent Modulus (E_tan_)
Magnitude	2680 kg/m^3^	69 GPa	0.33	292 MPa	690 MPa

**Table 3:** Material properties used in the FE model of rigid plate and bodies [19].

Material Properties	Mass Density (ρ)	Young’s Modulus (E)	Poisson’s Ratio (υ)
Magnitude	7830 kg/m^3^	207 GPa	0.34

**Table 4:** Comparison between FEA and experimental results at 5 ms^−1^.

Test Type	Honeycombs	Plateau Stress			Dissipated Energy		
		Exp.	FEA	Difference	Exp.	FEA	Difference
		MPa	MPa	%	J	J	%
**Indentation**	H31	1.23	1.17	4.88	382	364	4.71
**Indentation**	H42	2.13	1.89	11.26	644	571	11.33
**Compression**	H31	1.06	0.99	6.66	314	294	6.36
**Compression**	H42	1.64	1.45	11.58	499	441	11.62

**Table 5:** FEA results of honeycombs with constant cell wall thickness and different cell sizes.

Loading Type	FEA No.	Cell Size, D	Cell Wall Thickness, t	*t/l* Ratio	Plateau Stress	Dissipated Energy	Tearing Energy
		mm	mm	-	MPa	J	J
Indentation	CS-I-1	3.175	0.0254	0.01388	3.74	1262	309
	CS-I-2	3.969	0.0254	0.01109	2.81	955	224
	CS-I-3	4.763	0.0254	0.00924	2.18	731	183
	CS-I-4	6.35	0.0254	0.00692	1.39	472	153
	CS-I-5	9.525	0.0254	0.00462	0.73	256	78
Compression	CS-C-1	3.175	0.0254	0.01388	2.95	953	-
	CS-C-2	3.969	0.0254	0.01109	2.26	731	-
	CS-C-3	4.763	0.0254	0.00924	1.69	548	-
	CS-C-4	6.35	0.0254	0.00692	0.99	319	-
	CS-C-5	9.525	0.0254	0.00462	0.55	178	-

**Table 6:** FEA results of honeycombs with constant cell size and different cell wall thicknesses.

Loading Type	FEA No.	Cell Wall Thickness, t	*t/l* Ratio	Plateau Stress	Dissipated Energy	Tearing Energy
		mm	-	MPa	J	J
Indentation	TL-I-1	0.0178	0.00647	1.05	378	91
	TL-I-2	0.0254	0.00924	2.16	727	187
	TL-I-3	0.0381	0.01386	3.72	1260	315
	TL-I-4	0.0508	0.01847	4.91	1642	387
	TL-I-5	0.0635	0.02309	5.98	2005	449
	TL-I-6	0.0762	0.02771	7.24	2415	526
	TL-I-7	0.0889	0.03233	8.09	2695	678
	TL-I-8	0.1016	0.03695	9.21	3082	829
	TL-I-9	0.127	0.04618	11.39	3823	1064
	TL-I-10	0.1524	0.05542	13.24	4451	1370
Compression	TL-C-1	0.0178	0.00647	0.89	287	-
	TL-C-2	0.0254	0.00924	1.66	540	-
	TL-C-3	0.0381	0.01386	2.93	945	-
	TL-C-4	0.0508	0.01847	3.91	1255	-
	TL-C-5	0.0635	0.02309	4.82	1556	-
	TL-C-6	0.0762	0.02771	6.24	1889	-
	TL-C-7	0.0889	0.03233	6.65	2017	-
	TL-C-8	0.1016	0.03695	7.34	2253	-
	TL-C-9	0.127	0.04618	8.86	2759	-
	TL-C-10	0.1524	0.05542	9.86	3081	-
